# Determination of Phenol Compounds In Surface Water Matrices by Bar Adsorptive Microextraction-High Performance Liquid Chromatography-Diode Array Detection

**DOI:** 10.3390/molecules19079369

**Published:** 2014-07-03

**Authors:** Nuno R. Neng, José M. F. Nogueira

**Affiliations:** University of Lisbon, Faculty of Sciences, Chemistry and Biochemistry Department and Center of Chemistry and Biochemistry, Campo Grande Ed. C8, 1749-016 Lisbon, Portugal; E-Mail: ndneng@fc.ul.pt

**Keywords:** bisphenol-A, alkylphenol, nitrophenol, bar adsorptive microextraction, floating sampling technology, HPLC-DAD, surface waters

## Abstract

Bar adsorptive microextraction combined with liquid desorption followed by high performance liquid chromatography with diode array detection (BAµE-LD/HPLC-DAD) is proposed for the determination of trace levels of five phenol compounds (3-nitrophenol, 4-nitrophenol, bisphenol-A, 4-*n*-octylphenol and 4-*n*-nonylphenol) in surface water matrices. By using a polystyrene-divinylbenzene copolymer (PS-DVB) sorbent phase, high selectivity and efficiency is achieved even against polydimethylsiloxane through stir bar sorptive extraction. Assays performed by BAµE(PS-DVB)-LD/HPLC-DAD on 25 mL water samples spiked at the 10.0 µg/L levels yielded recoveries over 88.0% ± 5.7% for all five analytes, under optimized experimental conditions. The analytical performance showed good precision (RSD < 15%), detection limits of 0.25 µg/L and linear dynamic ranges (1.0–25.0 μg/L) with determination coefficient higher than 0.9904. By using the standard addition method, the application of the present method to surface water matrices allowed very good performances at the trace level. The proposed methodology proved to be a suitable alternative to monitor phenol compounds in surface water matrices, showing to be easy to implement, reliable, sensitive and requiring a low sample volume.

## 1. Introduction

Phenol compounds such as nitrophenols, bisphenols and alkylphenols are classified as potential endocrine disruptor chemicals and often detected in the environment [1]. These compounds are widely used as intermediates in industrial processes, namely in the production of explosives, pharmaceuticals, pesticides, resins, among others, and released into the environment in their original form or as by-products through waste or industrial discharges [2,3,4]. Due to the adverse effects on public health and the frequency at which they are detected in the environment, these compounds are listed as priority pollutants by the US and European environmental protection agencies [1]. Bisphenol-A (BPA), in particular, is one of the most produced compounds worldwide [5,6]. The major concern in the control of BPA is its use in the manufacture of materials that come into direct contact with foods, such as water bottles, cans, containers for food, among many others, which are the major sources of human exposure [7,8,9].

The most widely used methodologies for the determination of trace levels of phenol compounds is solid phase extraction followed by gas chromatography (GC) after derivatization [3,10]. Alternatively, solid phase microextraction (SPME) and stir bar sorptive extraction (SBSE) with *in-situ* derivatization followed by GC coupled to mass spectrometry (GC-MS) analysis, or LC-MS without the derivatization step had also been proposed to monitor the class of compounds [4,11,12,13,14,15]. Recently, our group has introduced a novel static microextraction technique, bar adsorptive microextraction (BAμE), that uses nanostructured materials, and have demonstrated it to be a remarkable alternative for trace analysis of medium-polarity to polar compounds in aqueous media [16,17]. This new analytical approach operates under the floating sampling technology and has demonstrated great advantages compared to other sorption-based methods (e.g., SBSE). Furthermore, BAμE allows one to tune the most convenient sorbent phase (e.g., activated carbons (ACs), polymers, *etc*.) for each particular target compound and has shown high effectiveness in many types of applications [18,19,20,21].

The present contribution aimed to apply BAµE followed by high performance liquid chromatography with diode array detection (HPLC-DAD) without derivatization for the determination of phenol compounds, including 3-nitrophenol (3NOP), 4-nitrophenol (4NOP), BPA, 4-*n*-octylphenol (OP) and 4-*n*-nonylphenol (NP) in water matrices. The optimization, validation, application and comparison with others sorbent-based techniques are also addressed.

## 2. Results and Discussion

### 2.1. Instrumental Conditions

In a first approach, the HPLC-DAD conditions including the UV/vis spectral data for the detection of five phenol compounds (3-nitrophenol, 4-nitrophenol, bisphenol-A, 4-*n*-octylphenol and 4-*n*-nonylphenol), as well as retention time characteristics were evaluated. In agreement with the UV/vis data obtained, the wavelength (λ_max_) of 226 nm was selected since it maximizes the detector’s response to the five analytes. By combining a conventional reversed phase column with a mobile phase constituted by MeOH and water, a good response was obtained for all analytes by HPLC-DAD, showing suitable resolution within convenient analytical time (<20 min). The instrumental sensitivity was checked through the limits of detection (LODs) and quantification (LOQs) for the analytes, obtained by the injection of diluted calibration standards and calculated with a signal-to-noise ratio (S/N) of 3/1 and 10/1, respectively. Values of 13.0 and 25.0 µg/L for LODs and LOQs, respectively, were obtained for 3NOP and for the remaining analytes under study (4NOP, BPA, OP and NP) the LODs and LOQs were 43.0 and 83.0 µg/L, respectively. Subsequently, the instrumental calibration was performed with six standards solutions having concentrations ranging from 0.1 to 4.0 mg/L. From the data obtained, excellent linear dynamic responses were achieved for all analytes with determination coefficients higher than 0.9944. To evaluate the instrumental precision, repeated injections for each calibration level were carried out, resulting in relative standard deviations (RSD) below 5.0%. No carry over was observed by a series of replicate injections since the background was always below the instrumental LODs achieved.

### 2.2. Selection of the Sorbent Phase

**Figure 1:**
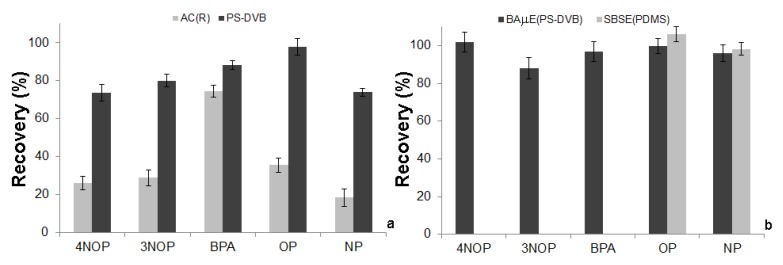
A Comparison of the recovery yields obtained by (**a**) BAµE using AC and PS-DVB as sorbent phases and (**b**) between BAµE (PS-DVB) and SBSE (PDMS) followed by LD/HPLC-DAD, under similar experimental conditions.

Before the method optimization, preliminary experiments were performed to select the most suitable sorbent phase for the determination of phenol compounds. For this purpose, a comparison between PS-DVB and AC was performed under similar experimental conditions (extraction: 16 h (1000 rpm); back-extraction: 45 min (ACN) under sonification). While the selectivity of both sorbent phases is pH dependent, AC is a porous solid material that retains the solutes through electrostatic and/or dispersive interactions, according to the textural adsorption properties, surface area and pore dimensions [20,21]. On the other hand, PS-DVB phase is a reversed phase type, retaining the analytes according to the particle size, surface area and mechanisms involved, *i.e.*, π-π, dipole-dipole, hydrogen bonding and ionic interactions [19]. The data obtained are depicted in Figure 1a, in which the efficiency yields obtained by the PS-DVB phase are much higher than for the AC one for the five phenol compounds under study. From the data obtained we can conclude that the reversed phase extraction type seems to be more effective, under similar experimental conditions. Consequently, PS-DVB was chosen as sorbent phase for the subsequent assays.

### 2.3. Optimization of the Recovery Assays

**Figure 2:**
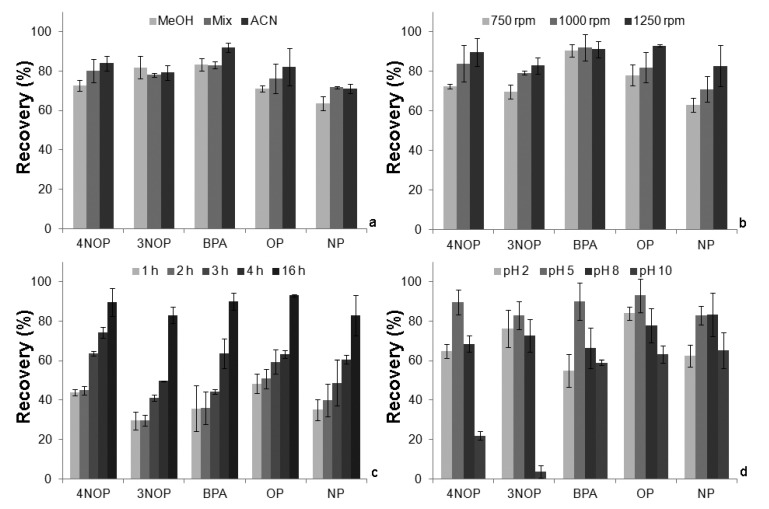
(**a**) Effect of desorption solvent, (**b**) agitation speed, (**c**) equilibrium time and (**d**) pH on the recovery yields.

For the present work, several parameters that affect the extraction and back-extraction efficiency yields were evaluated. Therefore, systematic assays were performed to optimize parameters such as, equilibrium time, agitation speed, matrix characteristics (pH, organic modifier and ionic strength) and LD conditions. In a first approach, the LD conditions that ensure complete back-extraction for five analytes from the PS-DVB phase were optimized. Solvents such as MeOH, ACN and a mixture (Mix) with equal volumes of both were assayed to survey the desorption performance, followed by solvent switch to a more suitable for HPLC-DAD analysis. Figure 2a depicts the data obtained (extraction time: 16 h (1000 rpm), pH 5.5 and back-extraction time: 45 min under sonification). From the data obtained, the ACN was selected as back-extraction solvent, since it presents the highest ability to desorpt the five phenols from the PS-DVB phase. After the selection of the most effective solvent for back-extraction, desorption times of 30, 45 and 60 min were also assayed, in which 45 min is enough and no advantages were obtained by using longer periods of time (data not shown). Consequently, 45 min was established for the back-extraction process.

According to the literature [18,19,20,21], the equilibrium time and stirring speed are extremely important parameters to be optimized for better extraction conditions. Concerning the stirring rate, it can have great influence in the mass transfer kinetics of the analytes towards to sorbent phase, as well as the floating sampling process. Thus, these two parameters had been evaluated for the PS-DVB devices extraction process. Figure 2b shows that 1,250 rpm gave a better performance on the recovery yields of the five analytes and was selected for further assays. Subsequently, the equilibrium time was carried out by experiments within 1, 2, 3, 4 and 16 h. As illustrated in Figure 2c, the recovery yields still increased after 16 h of the extraction. Although the extraction process is a bit slow, this analytical approach can be performed overnight. Thereby, the extraction time was set at 16 h for further experiments.

As demonstrated in previous works [18,19,20,21], the matrix characteristics, *i.e.*, pH, ionic strength and polarity are also important parameters that significantly affect the extraction efficiency. It must be emphasized that, for the phenol compounds, the pH plays an important role since these types of analytes are ionisable.

The results obtained are presented in Figure 2d, and show, as expected, that the pH influences the extraction process and plays an important role in the recovery yields. From the data obtained, it is possible to see that pH 5 is the best value for the interaction of phenols under study with the PS-DVB phase, whereas a more acidic or basic matrix media reduces the recovery yields since the analytes became charged, reducing therefore the selectivity.

The ionic strength and polarity were also modified through the addition of NaCl and MeOH (5%, 10% and 15%) to the matrix media, respectively. In a first approach, the addition of 5% NaCl increased slightly the recovery yields, whereas the progressive addition of MeOH decreased significantly the recovery yields of the five phenols under study (data not shown). As a consequence, the matrix pH 5 and 5% of NaCl were established for further experiments in the absence of MeOH.

### 2.4. Validation of BAµE(PS-DVB)-LD/HPLC-DAD Methodology

After the optimization study we proceed to the validation of the proposed methodology. From the data obtained, excellent linearity (1.0–25.0 µg/L) was obtained with remarkable determination coefficients (*r*^2^ > 0.9904).

**Table 1:** Recovery yields, determination coefficients (*r*^2^), LODs and LOQs achieved for five phenol compounds in ultrapure water samples using BAµE(PS-DVB)-LD/HPLC-DAD analysis, under optimized experimental conditions.

Phenol Compounds	Recovery ^a^ (% ± RSD; *n* = 3)	*r*^2 b^	LOD (µg/L)	LOQ (µg/L)
4NOP	102.2 ± 5.2	0.9995	0.3	0.8
3NOP	88.0 ± 5.7	0.9954	0.3	0.8
BPA	96.7 ± 5.3	0.9904	0.3	0.8
OP	99.8 ± 4.0	0.9946	0.3	0.8
NP	96.2 ± 4.5	0.9940	0.3	0.8

^a^ Method efficiency after extraction and back-extraction with PS-DVB in water sample spiked at the 10.0 μg/L level under optimized conditions (extraction: 16 h (1,250 rpm), pH 5, 5% NaCl; back-extraction: 45 min with ACN under sonification); ^b^ Seven levels of concentration ranging from 1.0 to 25.0 μg/L.

It is also noteworthy that the precision achieved for the present methodology, using within- and between-day repeatability assays calculated as relative standard deviation (RSD) on five replicates, gave rise to variations lower than 15.0%. It must be noted that, according to the requirements of Directive 98/83/EC for organic compounds, the present methodology may be considered acceptable since it presented a RSD below 25%.

The sensitivity of the actual methodology was also checked through the LODs and LOQs for all five compounds and calculated with a signal-to-noise ratio (S/N) of 3/1 and 10/1, respectively, where values of 0.3 µg/L for LODs and 0.8 µg/L for LOQs were achieved. The carry-over was also checked by a series of replicates, for which the background was always below the LODs achieved. Table 1 summarizes the experimental recovery yields, the determination coefficients, LODs and LOQs achieved for five phenols in ultra-pure water matrices by the present methodology.

### 2.5. Comparison between BAµE(PS-DVB) and SBSE(PDMS)

To demonstrate the advantages of the proposed analytical methodology (BAµE(PS-DVB)) over other static microextraction approaches, SBSE(PDMS) was evaluated under similar experimental conditions. Figure 1b depicts the comparison between BAµE(PS-DVB) and SBSE(PDMS), where must better performance can be observed with the former. The data obtained from both methodologies proved that the recovery yields obtained by BAµE(PS-DVB) method is higher than 80% to recover 4-NOP, 3-NOP and BPA, whereas SBSE(PDMS) is not efficient at all. Nevertheless for OP and NP both recoveries are similar since these two analytes present non-polar characteristics, evidenced by octanol-water partition coefficients (log *K*_O/W_) higher than 5.50, which is definitely in agreement with the SBSE(PDMS) theoretical predictions [22]. This observation reinforced the unsuitability of the PDMS polymeric phase for compounds with this type of polarity characteristics, present the limitation of SBSE to several groups of polar compounds. On opposition, BAµE shows great ability for trace level analysis of polar compounds, having the advantage for allowing to choose the most indicated sorbent phase for each particular type of application.

### 2.6. Application to Real Matrices

**Figure 3:**
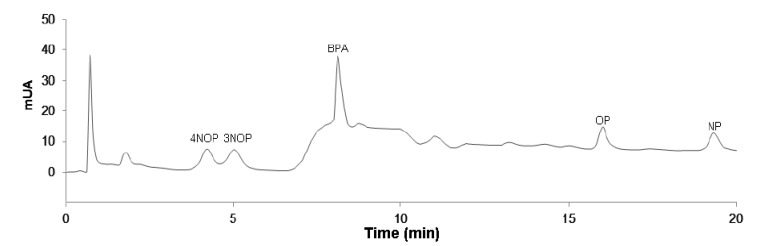
Chromatogram obtained from assays performed in a surface water spiked with 20 μg/L by BAµE(PS-DVB)-LD/HPLC-DAD, under optimized experimental conditions.

To evaluate the applicability of the proposed methodology, assays on three surface water matrices were performed. To account for intrinsic contamination and particular pronounced matrix effects, the standard addition method (SAM) was preferred instead of the conventional external calibration. In a first approach, the matrix was fortified with four working standards to produce the corresponding spiking levels (5.0–20.0 µg/L) for five compounds under study. Blank assays (“zero-point”) were also performed without spiking to assure maximum control of the analytical methodology. The results obtained from the assays performed by the SAM using the propose methodology, present an excellent selectivity and very good linearity with determination coefficient (*r*^2^) ranging from 0.9907 to 0.9959. The concentrations of the five phenol compounds under study were always below the LODs attained for all surface water matrices studied. It must be emphasized, according to EEC Drinking Water Guideline 80/779/EEC, No. L229/11-29, that in surface water matrices the maximum admissible values is between 1 and 3 µg/L. Therefore, the present methodology is suitable for the analysis of phenol compounds in surface waters, since the LODs achieved are below. Figure 3 exemplifies a chromatogram from a surface water sample spiked at the 20.0 µg/L, obtained by BAµE(PS-DVB)-LD/HPLC-DAD, under optimized experimental conditions, where good selectivity and sensitivity are notice. It must be emphasized that the present methodology, besides easy to work-up and environmentally friendly, exhibits a remarkable range of applicability, since the methodology promotes very high selectivity for phenol compounds, avoiding potential interferences and simultaneously a great analytical sensitivity at the trace level. In spite of the present methodology has proven to be a suitable tool to analyze phenols in surface waters at trace level, the performance can be further enlarged by using HPLC coupled to mass spectrometry or tandem systems (HPLC-MS or HPLC-MS/MS), in order to achieve even better analytical selectivity and sensitivity, lowering the LODs and helping in the identification of the analytes in complex matrices.

## 3. Experimental Section

### 3.1. Chemicals and Samples

All reagents and solvents were analytical grade and used with no further purification. The LiChrolut^®^ EN polymer sorbent (PS-DVB) for environmental analysis, HPLC-grade methanol (MeOH, 99.8%) and acetonitrile (ACN, 99.8%) were purchased from Merck (Darmstadt, Germany). Sodium chloride (NaCl, 99.9%) and sodium hydroxide (NaOH, 98.0%) were obtained from AnalaR BDH Chemicals (Darmstadt, Germany). 4-Nitrophenol (≥99%), 3-nitrophenol (99%), and bisphenol-A (99%) were purchased from Sigma-Aldrich (Munich, Germany). 4-*n*-Octylphenol (99.9%) and 4-*n*-nonylphenol (99.7%) were purchased from Supelco (Munich, Germany). The AC, hydrochloride acid (37.0%) and sodium carbonate (99.5%) were purchased from Riedel-de Haën (Munich, Germany). Ultra-pure water was obtained from Milli-Q water purification systems (Bedford, MA, USA). The surface water matrices were collected in the metropolitan area of Lisbon (Portugal) and were previously filtered (Whatman No. 1 filters) before their analysis.

### 3.2. Experimental Set-Up

#### 3.2.1. Preparation of the Microextraction Bars

The microextraction devices were prepared “in house” by coating polyethylene bars (15 mm length and 0.5 mm thickness) with adhesive films and then covered with PS-DVB (~5.0 mg) or AC (~1.5 mg) powders. The microextraction bars were previously cleaned by treatment with MeOH followed by ultrapure water before be used. The detailed description of the devices manufacturing can already be consulted [17].

#### 3.2.2. Recovery Assays and Method Validation

In a typical assay, ultra-pure water (25 mL, pH 5.5) spiked with a mixture of the five working standards at a desired concentrations, a microextraction bar device and conventional Teflon magnetic bar were introduced into a glass flask. For the optimization purposes assays were performed in a Variomag multipoint 15 agitation plate (Thermo Fisher Scientific Inc., Waltham, MA, USA) at room temperature. Parameters such as agitation speed (750, 1,000 and 1,250 rpm), extraction time (1, 2, 3, 4 and 16 h), matrix pH (2, 5, 8 and 10), organic modifier (MeOH; 5%, 10% and 15%, v/v) and ionic strength (NaCl; 5%, 10% and 15%, w/v) were systematically studied in triplicate. For back-extraction (LD), the microextraction bars were removed from the samples with clean tweezes and placed into a 2 mL vial containing 1.5 mL of the stripping solvent, ensuring their total immersion prior to ultrasonic treatment at a constant temperature (25 °C). To evaluate the best LD conditions, several assays using MeOH, ACN and a mixture of both (1:1) with different desorption times 30, 45 and 60 min) were performed in triplicate. After back-extraction, the microextraction bars were removed by clean tweezes, the stripping solvent was evaporated until dryness under a gentle stream of purified nitrogen (>99.5%), followed by reconstitution with 200 µL of mobile phase. The vials were then sealed and placed on the auto-sampler for HPLC-DAD analysis. For the method validation experiments, 25 mL of ultra-pure water were spiked with 200 µL of the working standard mixture at desired concentrations, and the extraction and back-extraction experiments were performed as described above under optimized conditions. For real sample assays, 25 mL of water sample and the standard addition methodology (SAM) were used, following by the same procedure employed for the validation experiments. Blank assays were also performed using the procedure above described without spiking.

### 3.3. Instrumentation Settings

HPLC-DAD analyses were carried out on an Agilent 1100 Series LC system (Agilent Technologies, Santa Clara, CA, USA), constituted by the following modules: vacuum degasser (G1322A), quaternary pump (G1311A), autosampler (G1313A), thermostatted column compartment (G1316A) and the diode array detector (G1315B). The data acquisition and instrumental control were performed by the software LC3D ChemStation (version Rev.A.10.02 [1757], Agilent Technologies). Analyses were performed on a Tracer excel 120 ODS-A column, 150 mm × 4.0 mm, 5 µm particle size (Teknokroma, Barcelona, Spain). The mobile phase consists on a mixture of MeOH (solvent A) and water with the following gradient: 0–4 min: 60% A, 4–10 min: 60%–90% A and 10–20 min: 90% A, using a flow of 0.5 mL/min. The injection volume was 20 μL with a draw speed of 200 μL min^−1^. The detector was set at 226 nm. For identification purposes, standard addition was used by spiking the samples with pure standards, as well as by comparing the retention parameters and peak purity with the UV/vis spectral reference data. For recovery calculations, peak areas obtained from each assay were compared with the peak areas of standard controls used for spiking. For quantification purposes on real matrices, calibration plots using the standard addition methodology was also performed.

## 4. Conclusions

The combination of bar adsorptive microextraction using PS-DVB sorbent phase and liquid desorption followed by high performance liquid chromatography with diode array detection was successfully applied to monitor five phenol compounds (4-nitrophenol, 3-nitrophenol, bisphenol-A, 4-*n*-octylphenol and 4-*n*-nonylphenol) in surface water matrices. Parameters affecting the extraction and back-extraction steps were fully optimized. By using PS-DVB phase, good accuracy, suitable precision, low detection limits and excellent linear range were achieved under optimized experimental conditions. By using the standard addition method, the application of the present analytical approach to surface water matrices provided very good performance at the trace level. The proposed method was also demonstrated to involve easy work-up, without derivatization steps, to be sensitive and require small amounts of low sample to monitor phenol compounds in water matrices. The methodology has proved to be a convenient alternative to monitor polar compounds at the trace level in comparison with other static microextraction techniques (e.g., SBSE(PDMS)), and has the advantage of being designed to support sorbents with different properties, allowing the study of their analytical performances, in the demand of searching for better enrichment techniques prior to instrumental analyses.
